# A Rapid Test to Differentiate Viral From Bacterial Infections: Searching for the Holy Grail

**DOI:** 10.1093/cid/ciaf585

**Published:** 2026-02-25

**Authors:** Lao-Tzu Allan-Blitz, Jeffrey D. Klausner

**Affiliations:** 1 Division of Global Health Equity: Department of Medicine, Brigham and Women’s Hospital, Boston, Massachusetts, USA;; 2 Department of Population and Public Health Sciences, Keck School of Medicine, University of Southern California, Los Angeles, California, USA

**Keywords:** point-of-care tests, antimicrobial stewardship, viral infections, bacterial infections

## Abstract

Differentiating viral from bacterial causes of acute respiratory illness can avoid the numerous consequences of inappropriate antibiotic use. We review the clinical literature evaluating a combined rapid Myxovirus resistance protein A and C-reactive protein point-of-care assay, discuss potential applications, and highlight remaining questions.

Acute respiratory infections led to over 13 million emergency department visits in the United States in 2022 [1]. More than 25% of such patients with bronchiolitis between 2007 and 2015 were treated with antibiotics, of which 70% had no documented bacterial infection [2]. Of antibiotics prescribed to privately insured patients with acute respiratory illnesses in 2016, 23% were inappropriate [3]. Inappropriate antibiotic use among children in the United States was associated with an increase in 7-day adverse events and an estimated $24.5 million in attributable expenditures (2025 US dollar) [4]. Exposure to antibiotics early in life may increase the risk for obesity and asthma [5, 6] as well as drive antimicrobial resistance—a major global public health threat [7]. Thus, effective strategies to assure the appropriate use of antimicrobials among cases of acute respiratory illness are urgently needed.

For decades, researchers have sought a means to distinguish bacterial from viral infections. C-reactive protein (CRP) has been used since its discovery in the 1930s. In the early 1990s, procalcitonin was introduced; however, while more accurate than CRP alone [8], procalcitonin was shown to be insufficiently sensitive for some use cases [9]. More recently, a score using CRP and 2 other cytokines demonstrated promising discrimination [10]. Yet, such tests are not widely available. We describe the biologic mechanisms of a point-of-care lateral flow assay combining CRP and Myxovirus resistance protein A (MxA) to distinguish bacterial from viral infections, highlight available clinical performance data, discuss potential applications, and consider areas for further research.

## C-REACTIVE PROTEIN AND MYXOVIRUS RESISTANCE PROTEIN A

CRP was discovered in the 1930s among patients with pneumococcal pneumonia. C-reactive protein is an acute phase reactant produced by the liver and stimulated predominantly by mediators of the innate immune system, specifically interleukin 6 [11]. C-reactive protein rises within 4–6 h of inflammation and peaks within 36 h [12]. C-reactive protein has been studied as a biomarker to identify numerous infectious states, with higher values (above 80 mg/L) thought to represent more severe infections [12]; however, CRP may be less accurate than procalcitonin for distinguishing bacterial from viral infections [8]. Yet, CRP is readily measured, making it an appealing biomarker for point-of-care assays.

Myxovirus proteins were discovered in the 1960s among mice with resistance to influenza viruses [13]. Related genes were later discovered in humans encoding MxA [14]. Myxovirus resistance protein A is expressed in response to type 1 interferon [15]—a mediator of the innate immune system’s specific response to viral infections [16], which does not rise in response to even severe bacterial infections [17]. Myxovirus resistance protein A is exclusively stimulated by interferon type 1 and not by pathogens or pathways of response to bacterial infection (eg, interleukin-1 or tumor necrosis factor alpha) [18]. *In vitro* studies have shown that levels of MxA rise within 1–2 h of interferon induction and have a half-life of approximately 2 days [19]. Mechanistically, MxA inhibits transcription and replication of a broad range of viruses [18].

Numerous studies have evaluated MxA as a marker of viral infection [20]. MxA levels were significantly higher among 95 children with viral infections determined by either polymerase chain reaction (PCR) testing or clinical judgement compared to 27 children with bacterial infections or 52 age-matched uninfected controls [21]. Among 247 children with symptoms of lower respiratory tract infection, 5 with bacterial infections, MxA detection had an area under the receiver operating characteristics curve of 90% for classifying viral versus bacterial etiology [22]. In a prospective cohort study of 193 children, MxA levels demonstrated a 96% sensitivity for distinguishing viral infections from bacterial infections and uninfected controls [23]. Those and other studies demonstrated that elevated MxA was associated with infection with RNA viruses such as influenza A and B, parainfluenza virus, respiratory syncytial virus, coronavirus, including SARS-CoV-2, and human metapneumovirus [23, 24], as well as with DNA viruses such as Epstein–Barr virus, herpes simplex virus types 1 and 2, cytomegalovirus, and adenovirus [24].

However, while a positive MxA test might indicate the presence of a viral infection, neither a positive nor a negative result excludes a bacterial infection. Simultaneous detection of a CRP value below 20 mg/L would likely indicate a viral infection, whereas a negative MxA result and detection of CRP above 20 mg/L [12] could indicate a non-viral illness—which in the right clinical context could warrant antibiotic therapy. The FebriDx^®^ assay (Lumos Diagnostics, United States) is a Food and Drug Administration–cleared test that incorporates qualitative detection of both MxA (≥40 ng/mL) and CRP (≥20 mg/L) into a disposable lateral flow device and provides results after 10 min.

## CLINICAL PERFORMANCE OF A COMBINED MYXOVIRUS RESISTANCE PROTEIN A/C-REACTIVE PROTEIN TEST

The MxA/CRP assay has been evaluated across numerous contexts (Table 1). A prospective single-center trial among 54 patients with acute, febrile respiratory tract illness found that the MxA/CRP assay correctly identified 80% of individuals with a bacterial infection confirmed with PCR, positive cultures, bacterial-specific urine antigens, or elevated bacterial-specific IgM antibodies [24]. Negative MxA/CRP results also correctly identified 92% of individuals with no infectious cause of their symptoms [24].

Another prospective multicenter study among 205 patients compared the MxA/CRP test to a reference testing algorithm that included throat culture, PCR testing from an upper respiratory tract sample, procalcitonin, white blood cell count, bandemia, and clinical judgement. That study found a 92% agreement for bacterial infection (sensitivity 80%, specificity 93%) [25]. The MxA/CRP assay demonstrated a 92% overall agreement with reference testing and/or physician panel adjudication in a subsequent prospective multicenter trial among patients with febrile upper respiratory tract infections [26]. Similar results were reported by a multicenter study of 520 patients presenting with febrile acute respiratory tract infections to outpatient centers; the MxA/CRP assay had a 93% sensitivity and 88% specificity for bacterial infections compared to a reference testing algorithm that included PCR and culture for 28 different pathogens as well as serum evaluation of procalcitonin, bacterial and viral antigens and white blood cell counts [27]. Finally, in a real-world study among adults, the MxA/CRP assay had an 87% sensitivity and 64% specificity for bacterial infection; of note, 93% of participants in that study had at least one comorbidity, nearly 30% were using immunosuppressive medications, and 30% were asymptomatic [28].

The MxA/CRP results also impact management. A single-center retrospective study in the primary care setting found that MxA/CRP testing led to deferral of antibiotics in 8 of 12 (67%) patients for whom bacterial respiratory tract infections were initially suspected [29]. Among 216 children with symptoms of an acute respiratory illness, MxA/CRP results led to a change in the therapeutic plan and a decision to defer antibiotics in 10% of cases [30]. Among 162 participants from 9 outpatient practices with symptoms of a lower respiratory tract infection deemed likely to receive antibiotics, MxA/CRP results led to a 41% reduction in antibiotic use with no increase in representation to care over 28 days of follow-up [31]. A cost analysis estimated that an MxA/CRP assay with a 95% sensitivity for bacterial infections would save over 2.5 billion dollars per year in the United States alone, with savings persisting even with a sensitivity of 75% [32].

The MxA/CRP test has also been used to triage patients. Among 1321 patients with symptoms of COVID-19, 84% were negative by MxA/CRP, of which 78% were triaged to a low-risk area leading to a significant reduction in use of emergency department resources [33]. Elsewhere, MxA/CRP results permitted 826 patients with suspected COVID-19 to be released from quarantine who would have required quarantine based on clinical criteria [34]. Notably, in that study MxA/CRP results missed 10 of 136 cases of SARS-CoV-2 infection detected by PCR, which were either mildly symptomatic or asymptomatic.

## REMAINING QUESTIONS AND FUTURE DIRECTIONS

While available performance data are promising, additional questions remain. How MxA/CRP testing performs in other settings, such as intensive care units or inpatient wards, as well as for less common viruses is not known. Similarly, viral evolution might alter the immune response. One study found that the MxA/CRP assay had a notably lower sensitivity for viral infections (56%) during a period when a novel strain of SARS-CoV-2 was the predominant circulating variant [35].

Further, no gold standard diagnostic exists that can distinguish bacterial from viral infections against which to compare the MxA/CRP results. Molecular assays have high sensitivity for specific pathogens; however, currently available diagnostics are not sufficiently robust to definitively rule out all viral or bacterial causes of respiratory illnesses. Thus, individual study-level performance data should be interpreted within the context of the existing diagnostic limitations.

Additionally, more than 3% of adults and up to 15% of children with bacterial pneumonia may have a detectable viral pathogen [36, 37]. In such instances, positive MxA and CRP results may lead to inappropriate discontinuation or deferral of antibiotics in the absence of other clinical manifestations of a bacterial infection. Similarly, some viral infections have been shown to raise CRP values above 100 mg/L [38], which will likely still result in inappropriate antibiotic use among a minority of cases. Furthermore, CRP peaks after 36 h, and thus false-negative values may occur early in the disease course. For those reasons, clinical context and follow-up remain essential; however, those considerations do not necessarily negate the impact of rapid MxA/CRP testing. Further research is needed to delineate the limitations of the test. Additionally, more evidence is needed on clinical outcomes for patients treated based on results of the MxA/CRP assay.

Thus far, the MxA/CRP assay has been utilized predominantly in emergency department and primary care settings. Additional areas that may benefit from such tests include nursing homes and long-term care facilities. More than half of residents in some long-term care settings may be prescribed antibiotics within a 12 month period [39]. Similarly, antibiotics are prescribed in nearly 40% of urgent care visits and nearly half of inappropriate antibiotic prescriptions occur in urgent care centers [40]. Thus, substantial benefits from antimicrobial stewardship may be appreciated if rapid MxA/CRP testing is introduced in such settings. However, further research is needed to confirm that the test performs as expected in other settings and among other populations.

## CONCLUSIONS

Combined MxA/CRP testing can reliably exclude bacterial infections in acute respiratory illness. The high negative predictive value provides confidence for clinical decision making. However, important questions remain, including delineating longitudinal clinical outcomes. Along with other rapid point-of-care tests to detect specific pathogens, the MxA/CRP test may finally provide a way for frontline clinicians to objectively determine the need for antimicrobials among cases of acute respiratory illnesses, the importance of which cannot be overstated.

## Figures and Tables

**Table 1:** Studies Evaluating the Performance of a Combined Point-of-Care Myxovirus Resistance Protein A and C-Reactive Protein Assay for Detecting Bacterial Infections

							Predicting Bacterial Infection^c^		
Year	Author	Country	No. of Participants	Clinical Presentation	Age in Years (Mean)	Reference Testing Method	Negative Predictive Value (95% CI)	Positive Predictive Value (95% CI)	Ref.
2015	Sambursky	United States	54	Febrile pharyngitis Febrile lower respiratory symptoms	18–69 (37) 22–29 (51)	PCR, bacterial culture, urine antigen, chest X-ray	80% (56%−94%)	88% (73%−97%)	[24]
2017	Self	United States	205	Febrile upper respiratory tract symptoms	Median 29 (IQR 31)	PCR, antibody serology, bacterial culture, procalcitonin, white blood cell count	97% (94%−99%)	63% (45%−79%)	[25]
2018	Shapiro	United States	220	Febrile upper respiratory tract symptoms	2–86 (37)	PCR, antibody serology, bacterial culture, procalcitonin, white blood cell count	99% (93%−100%)	76% (59%−87%)	[26]
2022	Shapiro	United States	520	Recently (within 3 d) febrile with acute respiratory symptoms^a^	1 to >65 (35)	PCR, antibody serology, bacterial culture, procalcitonin, white blood cell count	99% (97%−99%)	58% (49%−67%)	[27]
2023	Tong-Minh	Netherlands	224	Individuals with respiratory symptoms or who had throat swabs collected	Median 58 (IQR 28)	PCR, bacterial culture, procalcitonin, chest imaging^b^	89% (82%−94%)	60% (51 %−68%)	[23]

Abbreviations: CI, confidence interval; IQR, interquartile range; PCR, polymerase chain reaction; Ref., reference.

a Acute respiratory symptoms were defined as at least 1 of 6 of the following: rhinorrhea, nasal congestion, sore throat, cough, hoarseness, or shortness of breath.

b Chest imaging included computed tomography scans or lung ultrasound.

c Sensitivity for bacterial infections ranged from 80%–93%, while specificity for bacterial infections ranged from 67%–93%.

## References

[1] Centers for Disease Control and Prevention. National center for health statistics: national hospital ambulatory medical care survey: 2022 emergency department summary tables. 2022. Available at: https://www.cdc.gov/nchs/data/nhamcs/web_tables/2022-nhamcs-ed-web-tables.pdf. Accessed 12 June 2025.

[2] Papenburg J, Fontela PS, Freitas RR, Burstein B. Inappropriate antibiotic prescribing for acute bronchiolitis in US emergency departments, 2007–2015. J Pediatric Infect Dis Soc 2019; 8:567–70. 30657968 10.1093/jpids/piy131

[3] Chua KP, Fischer MA, Linder JA. Appropriateness of outpatient antibiotic prescribing among privately insured US patients: ICD-10-CM based cross sectional study. BMJ 2019; 364:k5092. 30651273 10.1136/bmj.k5092 PMC6334180

[4] Butler AM, Brown DS, Durkin MJ, Association of inappropriate outpatient pediatric antibiotic prescriptions with adverse drug events and health care expenditures. JAMA Netw Open 2022; 5:e2214153. 35616940 10.1001/jamanetworkopen.2022.14153 PMC9136626

[5] Cox LM, Blaser MJ. Antibiotics in early life and obesity. Nat Rev Endocrinol 2015; 11:182–90. 25488483 10.1038/nrendo.2014.210 PMC4487629

[6] Murk W, Risnes KR, Bracken MB. Prenatal or early-life exposure to antibiotics and risk of childhood asthma: a systematic review. Pediatrics 2011; 127: 1125–38. 21606151 10.1542/peds.2010-2092

[7] Laxminarayan R, Matsoso P, Pant S, Access to effective antimicrobials: a worldwide challenge. Lancet 2016; 387:168–75. 26603918 10.1016/S0140-6736(15)00474-2

[8] Norman-Bruce H, Umana E, Mills C, Diagnostic test accuracy of procalcitonin and C-reactive protein for predicting invasive and serious bacterial infections in young febrile infants: a systematic review and meta-analysis. Lancet Child Adolesc Health 2024; 8:358–68. 38499017 10.1016/S2352-4642(24)00021-X

[9] Kamat IS, Ramachandran V, Eswaran H, Guffey D, Musher DM. Procalcitonin to distinguish viral from bacterial pneumonia: a systematic review and meta-analysis. Clin Infect Dis 2020; 70:538–42. 31241140 10.1093/cid/ciz545

[10] Fröhlich F, Gronwald B, Bay J, Expression of TRAIL, IP-10, and CRP in children with suspected COVID-19 and real-life impact of a computational signature on clinical decision-making: a prospective cohort study. Infection 2023; 51:1349–56. 36757525 10.1007/s15010-023-01993-1 PMC9910257

[11] Zhou HH, Tang YL, Xu TH, Cheng B. C-reactive protein: structure, function, regulation, and role in clinical diseases. Front Immunol 2024; 15: 1425168. 38947332 10.3389/fimmu.2024.1425168 PMC11211361

[12] Markic J, Saraga M, Dahlem P. Sepsis biomarkers in neonates and children: C-reactive protein and procalcitonin. J Child Sci 2017; 7:e89–95.

[13] Lindenmann J Resistance of mice to mouse-adapted influenza A virus. Virology 1962; 16:203–4. 14465449 10.1016/0042-6822(62)90297-0

[14] Horisberger MA, Wathelet M, Szpirer J, cDNA cloning and assignment to chromosome 21 of IFI-78K gene, the human equivalent of murine Mx gene. Somat Cell Mol Genet 1988; 14:123–31. 3162334 10.1007/BF01534397

[15] Horisberger MA. Interferon-induced human protein MxA is a GTPase which binds transiently to cellular proteins. J Virol 1992; 66:4705–9. 1629950 10.1128/jvi.66.8.4705-4709.1992 PMC241296

[16] Baum A, García-Sastre A. Induction of type I interferon by RNA viruses: cellular receptors and their substrates. Amino Acids 2010; 38:1283–99. 19882216 10.1007/s00726-009-0374-0 PMC2860555

[17] Girardin E, Grau GE, Dayer JM, Roux-Lombard P, Lambert PH. Tumor necrosis factor and interleukin-1 in the serum of children with severe infectious purpura. N Engl J Med 1988; 319: 397–400. 3135497 10.1056/NEJM198808183190703

[18] Haller O, Kochs G. Human MxA protein: an interferon-induced dynamin-like GTPase with broad antiviral activity. J Interferon Cytokine Res 2011; 31:79–87. 21166595 10.1089/jir.2010.0076

[19] Ronni T, Melén K, Malygin A, Julkunen I. Control of IFN-inducible MxA gene expression in human cells. J Immunol 1993; 150:1715–26. 7679692

[20] Halminen M, Ilonen J, Julkunen I, Ruuskanen O, Simell O, Mäkelä MJ. Expression of MxA protein in blood lymphocytes discriminates between viral and bacterial infections in febrile children. Pediatr Res 1997; 41:647–50. 9128286 10.1203/00006450-199705000-00008

[21] Nakabayashi M, Adachi Y, Itazawa T, MxA-based recognition of viral illness in febrile children by a whole blood assay. Pediatr Res 2006; 60:770–4. 17065575 10.1203/01.pdr.0000246098.65888.5b

[22] Rhedin S, Eklundh A, Ryd-Rinder M, Myxovirus resistance protein A for discriminating between viral and bacterial lower respiratory tract infections in children—the TREND study. Clin Microbiol Infect 2022; 28:1251–7. 35597507 10.1016/j.cmi.2022.05.008

[23] Tong-Minh K, van Hooijdonk S, Versnel MA, Blood myxovirus resistance protein-1 measurement in the diagnostic work-up of suspected COVID-19 infection in the emergency department. Immun Inflamm Dis 2022; 10:e609. 35349755 10.1002/iid3.609 PMC8962640

[24] Sambursky R, Shapiro N. Evaluation of a combined MxA and CRP point-of-care immunoassay to identify viral and/or bacterial immune response in patients with acute febrile respiratory infection. Eur Clin Respir J 2015; 2:28245. 26672961 10.3402/ecrj.v2.28245 PMC4676840

[25] Self WH, Rosen J, Sharp SC, Diagnostic accuracy of FebriDx: a rapid test to detect immune responses to viral and bacterial upper respiratory infections. J Clin Med 2017; 6:94. 28991170 10.3390/jcm6100094 PMC5664009

[26] Shapiro NI, Self WH, Rosen J, A prospective, multi-centre US clinical trial to determine accuracy of FebriDx point-of-care testing for acute upper respiratory infections with and without a confirmed fever. Ann Med 2018; 50:420–9. 29775092 10.1080/07853890.2018.1474002

[27] Shapiro NI, Filbin MR, Hou PC, Diagnostic accuracy of a bacterial and viral biomarker point-of-care test in the outpatient setting. JAMA Netw Open 2022; 5:e2234588. 36255727 10.1001/jamanetworkopen.2022.34588 PMC9579916

[28] Tong-Minh K, Daenen K, Endeman H, Performance of the FebriDx rapid point-of-care test for differentiating bacterial and viral respiratory tract infections in patients with a suspected respiratory tract infection in the emergency department. J Clin Med 2023; 13:163. 38202172 10.3390/jcm13010163 PMC10779507

[29] Davidson M Febridx point-of-care testing to guide antibiotic therapy for acute respiratory tract infection in UK primary care: a retrospective outcome analysis. J Infect Dis Preve Med 2017; 5:165.

[30] de la Matta Farrando P, Suay Torres MT, Sabater Sabate A, Trenchs Sainz de la Maza V, Luaces Cubells C, Hernandez Bou S. Evaluation of FebriDx(R) for the management of children with acute febrile respiratory infection. Enferm Infecc Microbiol Clin (Engl Ed) 2024; 42:313–6. 38688819 10.1016/j.eimce.2024.04.002

[31] Wilcox CR, Odeh N, Clark TW, Use of the FebriDx(R) host-response point-of-care test may reduce antibiotic use for respiratory tract infections in primary care: a mixed-methods feasibility study. J Antimicrob Chemother 2024; 79:1441–9. 38708643 10.1093/jac/dkae127 PMC11144485

[32] Dick K, Schneider J. Economic evaluation of FebriDx(R): a novel rapid, point-of-care test for differentiation of viral versus bacterial acute respiratory infection in the United States. J Health Econ Outcomes Res 2021; 8:56–62. 34703832 10.36469/001c.27753 PMC8483888

[33] Mansbridge CT, Tanner AR, Beard KR, Febridx host response point-of-care testing improves patient triage for coronavirus disease 2019 (COVID-19) in the emergency department. Infect Control Hosp Epidemiol 2022; 43:979–86. 35094739 10.1017/ice.2021.531 PMC8828393

[34] Houston H, Deas G, Naik S, Utility of the FebriDx point-of-care assay in supporting a triage algorithm for medical admissions with possible COVID-19: an observational cohort study. BMJ Open 2021; 11:e049179. 10.1136/bmjopen-2021-049179 PMC8354759 34373308

[35] Buntine P, Miller J, Pope A, Negative predictive value of the FebriDx host response point-of-care test in patients presenting to a single Australian emergency department with suspected COVID-19: an observational diagnostic accuracy study. BMJ Open 2022; 12:e065568. 10.1136/bmjopen-2022-065568 PMC9805821 36581427

[36] Jain S, Self WH, Wunderink RG, Community-acquired pneumonia requiring hospitalization among U.S. adults. N Engl J Med 2015; 373:415–27. 26172429 10.1056/NEJMoa1500245 PMC4728150

[37] Jain S, Williams DJ, Arnold SR, Community-acquired pneumonia requiring hospitalization among U.S. children. N Engl J Med 2015; 372:835–45. 25714161 10.1056/NEJMoa1405870 PMC4697461

[38] Salonen EM, Vaheri A. C-reactive protein in acute viral infections. J Med Virol 1981; 8:161–7. 6276502 10.1002/jmv.1890080302

[39] Thornley T, Ashiru-Oredope D, Normington A, Beech E, Howard P. Antibiotic prescribing for residents in long-term-care facilities across the UK. J Antimicrob Chemother 2019; 74:1447–51. 30698718 10.1093/jac/dkz008 PMC6477989

[40] Palms DL, Hicks LA, Bartoces M, Comparison of antibiotic prescribing in retail clinics, urgent care centers, emergency departments, and traditional ambulatory care settings in the United States. JAMA Intern Med 2018; 178:1267–9. 30014128 10.1001/jamainternmed.2018.1632 PMC6142958

